# Association between ankle brachial index and development of postoperative intensive care unit delirium in patients with peripheral arterial disease

**DOI:** 10.1038/s41598-021-91990-x

**Published:** 2021-06-17

**Authors:** Jihee Kang, Ji Hyun An, Hong Jin Jeon, Yang Jin Park

**Affiliations:** 1 Division of Vascular Surgery, Department of Surgery, Inha University Hospital, Inha University School of Medicine, #27 Inhang-ro, Joong-gu, Incheon, 22332 Korea; 2 Department of Psychiatry, Depression Center, Samsung Medical Center, Sungkyunkwan University School of Medicine, #81 Irwon-ro, Gangnam-gu, Seoul, 06351 Korea; 3 Department of Health Sciences & Technology, Department of Medical Device Management & Research, and Department of Clinical Research Design & Evaluation, Samsung Advanced Institute for Health Sciences & Technology (SAIHST), Sungkyunkwan University, #81 Irwon-ro, Gangnam-gu, Seoul, 06351 Korea; 4 Division of Vascular Surgery, Department of Surgery, Samsung Medical Center, Sungkyunkwan University School of Medicine, #81 Irwon-ro, Gangnam-gu, Seoul, 06351 Korea

**Keywords:** Medical research, Risk factors

## Abstract

Patients with vascular diseases are prone to developing postoperative delirium (POD). Ankle brachial index (ABI) is a non-invasive clinical indicator of lower-extremities peripheral arterial disease (PAD) and has been identified as an indicator of cognitive impairment. We investigated the association between ABI and POD. 683 PAD patients who underwent elective leg arterial bypass surgery between October 1998 and August 2019 were collected for retrospective analysis. Demographic information, comorbidities, preoperative ABI and the Rutherford classification within one month prior to surgery were obtained. POD was assessed using the Confusion assessment method -intensive care unit. Logistic regression and receiver operating characteristics (ROC) curve analysis were used to assess the association between ABI and POD. The mean value of ABI was significantly lower in patients with POD than it was those without POD. Older age, more medical comorbidities, longer length of surgery, decreased ABI, and higher Rutherford class were all significantly associated with POD. The area under ROC (0.74) revealed that ABI below 0.35 was associated with development of POD. Lower preoperative ABI was associated with POD in PAD patients who underwent arterial bypass surgery.

## Introduction

Delirium is a neuropsychiatric disorder that is characterized by acute and fluctuating changes in baseline attention, awareness, and cognition^1^. Postoperative delirium (POD) is defined by a disturbance of consciousness that is accompanied by impaired attention or inability to focus that cannot be explained by a pre-existing or evolving neurocognitive disorder^1,2^. POD is well known to be associated with prolonged hospital stay and medical morbidities. It also increases medical costs^2–4^.

Previous literature has suggested that the incidence of POD in vascular patients ranges from 5 to 39%^4–6^. Furthermore, as life expectancy of the general population increases, the number of vascular patients will also inevitably increase, because aging is an important contributor to vascular disease^7^. A recent report on the global prevalence of peripheral arterial disease (PAD)^8^ showed that the prevalence of PAD increased consistently with age, and became a serious public health issue.

The risk factors of POD after vascular surgeries have been studied previously, and include the following: hypertension, history of cognitive impairment, history of delirium, open aortic surgery, major amputation surgery, and preoperative anemia^2,4,9,10^. These multifactorial aspects of POD make its management difficult. Therefore, efforts have been made to identify the predictive markers of POD. However, they were mostly biochemical markers that are not thought to be disease specific.

Ankle-brachial index (ABI) is a non-invasive, simple, and routinely used test to diagnose and follow lower-extremity PAD, with a sensitivity of 90% and a specificity of 98% if the value is less than 0.9^11,12^. Guerchet et al. suggested that a low ABI can be considered a marker of cognitive impairment^11^. According to a study to validate the increased rate of POD in patients with clinically diagnosed cognitive impairment or dementia at the time of surgery, cognitive impairment or dementia is a risk factor for POD^13^. POD affected not only short-term cognitive dysfunction, but also long-term cognitive impairment in a prospective study of 200 patients who underwent hip surgery^14^.

ABI has been also suggested to be a predictor of cardiovascular disease by other investigators^15,16^. We hypothesized that ABI is associated with development of POD, especially in patients who underwent leg arterial bypass surgery due to PAD. We also assumed that there is a cutoff value of ABI, since patients with PAD present with an abnormal ABI range. In this study, we tried to evaluate the association between ABI and POD in patients who underwent leg arterial bypass surgery due to PAD.

## Material and methods

This study was approved by the institutional review board (IRB) of Samsung Medical Center in Seoul, Korea. Obtaining informed consent was waived by the Ethics committee due to retrospective nature of the study. This retrospective chart review study involving human participants was in accordance with the ethical standards of the institutional and national research committee and with 1964 Helsinki Declaration and its later amendments or comparable ethical standards. In our institution, all leg arterial bypass surgery is performed under general anesthesia. Also, every patient who undergoes leg arterial bypass surgery is routinely transferred to the surgical ICU immediately following bypass surgery, and moved to general ward on the first postoperative day unless critical complications occur.

### Patient selection

**Figure 1:**
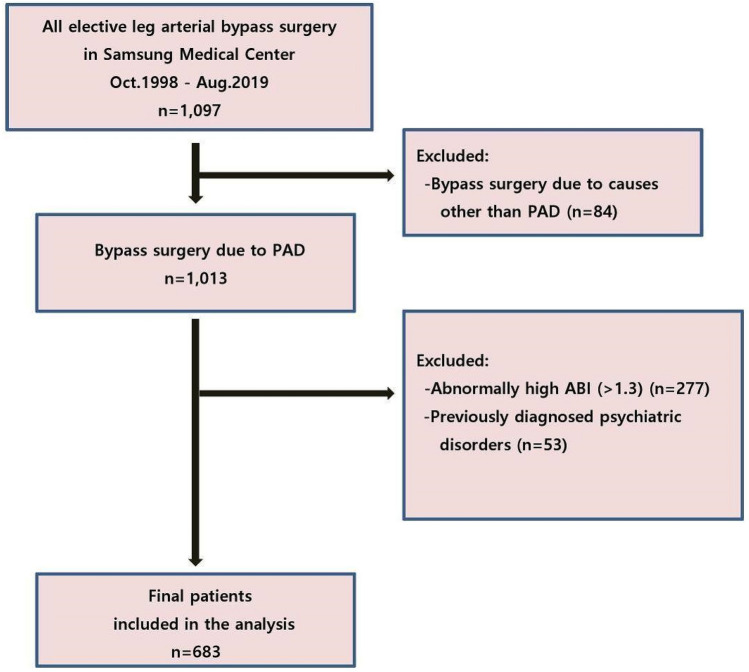
Patient selection flow diagram.

To select patients for retrospective analysis, we used the following institutional codes for surgery type to collect patients from the initial screening of the database: femoro-femoro bypass, femoro-popliteal bypass, femoro-tibial or -peroneal bypass, and popliteo-tibial or –peroneal bypass. We initially collected 1,097 patients who underwent elective leg arterial bypass surgery between October 1998 and August 2019 at Samsung Medical Center in Korea. Among 1,097 patients, we excluded patients who underwent elective leg arterial bypass surgery due to reasons other than lower-extremity PAD (N = 84). Patients who presented with an abnormally high ABI (> 1.3) due to calcified incompressible arteries^17^ were also excluded (N = 277). Falsely high ABI values do not correctly represent the status of peripheral arterial disease. More importantly, abnormally high ABI does not necessarily mean impairment of luminal patency nor presence of peripheral arterial disease^18^. Non-compressible leg arteries are common among patients with long-standing diabetes mellitus, chronic kidney disease, and also obesity^19^. In addition, patients with underlying bipolar affective disorder, schizophrenia, other psychotic disorders, substance or alcohol use disorders, mental disorders due to organic causes such as brain tumor, intellectual disability, dementia or neurological illness (including epilepsy) were also excluded (N = 53). Finally, a total of 683 patients were enrolled in the study (Fig. 1). Of note, 109 among 683 patients underwent more than one operation per admission. Considering the potential influence of additional operation and associated long procedural time to development of POD, we only included the first operation for each patient.

### Clinical assessment

The following clinical parameters were obtained from each patient at the initial visit: demographic information (age, sex, occupational status by current employment, total duration of education), lifestyle data (smoking, alcohol consumption), medical comorbidities, psychiatric comorbidities, Charlson comorbidity index (CCI) and American Society of Anesthesiologists (ASA) score. Three patients with history of heavy alcohol use as binge drinking on 5 or more days in the past month were excluded, since alcohol withdrawal would be a major contributing cause of POD. Of those three patients, one was in the abnormally high ABI group, and the other two were in the group with bypass surgery due to causes other than PAD. In addition, a qualified health professional assessed the severity grade according to the Rutherford classification within 1 month of surgery (with regard to lower-extremity PAD). Postoperatively, we also collected the following information: operative length, length of postoperative stay in the ICU, length of hospital stay, and mortality during the follow-up period. Identifying death was carried out using Korean Death and Causes of Death Statistics that is linked to electronic medical records.

### ABI measurements

ABI is defined by the ratio of the systolic blood pressure in the ankle to that in the brachial artery. The ABI is calculated according to the Trans-Atlantic Inter-Society Consensus Document on Management of Peripheral Arterial Disease II (TASC-II) guidelines^20^. The ABI is calculated by dividing the highest systolic blood pressure measured in each limb (anterior tibial artery or posterior tibial artery) to the highest systolic blood pressure measured in the right or left brachial artery^12^. An ABI < 0.90 is typically accepted to define PAD^11^. Normal values range between 1.0 and 1.3, while higher values occur when the arteries are incompressible due to heavy calcification^17^. At our institution, ABI, duplex ultrasonography and computed tomographic angiography (CTA) are routinely performed in every patient who is planned to undergo leg arterial bypass surgery. The ABI is measured using the volume-plethysmographic apparatus (Multilab Series II LHS, Unetixs Vascular Inc., RI, USA).

Assessment of POD. The study patients were assessed for POD with the CAM-ICU^21,22^, which includes the following four features: 1. Acute onset or fluctuating course; 2. Inattention; 3. Disorganized thinking; and 4. Altered level of consciousness. A patient who is ‘delirium positive’ has a positive result in the first two features, and in either feature 3 or 4, plus a Richmond Agitation-Sedation Scale (RASS)^23^ ≥  − 3. The Confusion Assessment Method (CAM) was created to better identify delirium in 1990, and it has become the most widely used standardized instrument to assess delirium due to its accuracy and ease of use^24^. The CAM-ICU is an adaptation of the CAM, developed by Inouye in 1990, and it is one of two monitoring scales recommended by the Society of Critical Care Medicine’s Clinical Practice Guidelines for the Management for delirium in adults patients in an ICU^24,25^. Trained nurses independently performed the confusion assessment method (CAM)–ICU once per their 8-h-duty. To preclude remaining effects of anesthetics and opioid, the very first CAM-ICU score upon ICU admission was excluded. The average observed CAM-ICU-7 scores during ICU days were used for delirium severity^26,27^.

A psychiatrist assessed ICU delirium according to the DSM-IV diagnostic criteria. The severity of POD was measured using the CAM-ICU-7 delirium severity scale^28^, which is a 7-point rating scale (0–7) that was adapted from the CAM-ICU and RASS. Each symptom of POD, except item 1 (which is acute onset or fluctuating course) was characterized on a scale from 0 (absent), to 1 (mild), to 2 (severe). In contrast, item 1 was a binary outcome with a result of 0 (absent) or 1 (present). The sum of the ratings was categorized as follows: 0–2: no POD; 3–5: mild to moderate; or 6–7: severe.

### Statistical analysis

The sociodemographic variables, preoperative measurements and postoperative outcomes were calculated by covariance analysis according to the severity of POD. We used logistic regression analyses to calculate the odds ratios (OR) of ABI with POD development after adjusting for age, sex, educational level, smoking and alcohol consumption. The variables with statistical significance in univariate analysis were entered into the multivariate analysis. Receiver operating characteristics (ROC) analysis was used to estimate the sensitivity and specificity of the ABI for assessing the association with POD. The cutoff value was determined to minimize the sum of false-positive and false-negative test results. Also, a high area under the curve (AUC) was presented. The results of logistic regression and the cutoff value of ABI were internally validated using the bootstrapping method with 1,000 replicates. All statistical analyses in this study were performed using IBM SPSS Statistics Software Version 21.0 (IBM, Armonk, NY, USA). A statistical significance cutoff was set at an alpha level of 0.05.

## Results

### Demographic characteristics and comorbidities of the patients

Table 1 presents the demographic characteristics and comorbidities of all study patients according to the severity of POD. The overall incidence of POD was 18.3%. The mean age was 66.18 ± 0.45 (mean ± SE) years in the no POD group, 70.19 ± 1.01 years in the mild to moderate group, and 72.75 ± 2.72 years in the severe group. Patients with POD were significantly older than were those without POD group, regardless of severity. Patients with POD were less likely to have a job than were patients without POD.

**Table 1:** Demographic characteristics and comorbidities according to POD development.

Characteristics	No POD (n = 558)	Mild to moderate (n = 110)	Severe (n = 15)	F or χ^2^ (*P-*value)	Post-hoc
Male sex, n (%)	498 (89.2)	93 (84.5)	13 (86.7)	2.033 (.36)	ns
Age at time of registration mean ± SE, y	66.18 ± 0.45	70.19 ± 1.01	72.75 ± 2.72	8.95 (.000)***	Mild to moderate, severe > no POD
Occupational status, n (%)	203 (37.1)	77.3 (22.7)	3 (23.1)	9.15 (.01)*	No POD > mild to moderate, severe
Education, mean ± SE, y	11.99 ± 2.71	11.55 ± 3.22	10.12 ± 0.39	2.51 (.11)	ns
Smoking, n (%)	459 (82.3)	85 (77.3)	12 (80.0)	1.52 (.47)	ns
CCI, mean ± SE	2.17 ± 0.06	2.58 ± 0.14	2.84 ± 0.40	4.86 (.008)**	Mild to moderate, severe > no POD
CCI, n (%)				11.54 (.07)	ns
0	90 (16.1)	12 (10.9)	1 (6.7)		
1	99 (17.7)	14 (12.7)	0 (0)		
2	145 (26.0)	26 (23.6)	6 (40.0)		
≥ 3	224 (40.1)	58 (52.7)	8 (53.3)		
ASA score, mean ± SE	2.43 ± 0.02	2.54 ± 0.05	2.86 ± 0.15	5.44(.005)**	Severe > no POD, mild to moderate
ASA score, n (%)				63.72(.000)***	Severe > no POD, mild to moderate
1	13 (2.3)	1 (0.9)	0 (0)		
2	298 (53.4)	46 (41.8)	3 (20.0)		
3	233 (41.8)	59 (53.6)	7 (46.7)		
4	9 (1.6)	3 (2.7)	5 (33.3)		

*POD* postoperative delirium, *SD* standard deviation, *SE* standard error, *CCI* Charlson comorbidity index, *ASA* American Society of Anesthesiologists. *P < .05, **P < .01, ***P < .001.

With regard to medical comorbidities, patients with mild to moderate POD (2.58) or severe POD (2.84) had a statistically higher mean CCI score than did those without POD (2.17) (*P* = 0.008). The severe POD group (2.86) also reported a significantly higher mean ASA score than did the no POD group (2.54) and the mild to moderate POD group (2.43) (*P* = 0.005). Furthermore, more patients with severe POD were categorized as higher ASA scores (of 3 and 4) than were those with mild to moderate POD and without POD (*P* = 0.000).

### Preoperative and postoperative measurements of PAD according to the POD severity

We compared the following parameters between the groups: preoperative ABI, preoperative Rutherford class, POD duration, mean CAM-ICU score, length of surgery, length of ICU stay, length of hospital stay and mortality in the follow-up period (Table 2).

**Table 2:** Preoperative PAD measurements and postoperative outcomes.

	No POD (n = 558)	Mild to moderate (n = 110)	Severe (n = 15)	F or χ^2^ (*P-*value)	Post-hoc
**1. Preoperative measurements**					
ABI, mean ± SE	0.52 ± 0.01	0.45 ± 0.02	0.29 ± 0.06	10.95 (.000)***	Severe > mild to moderate > no POD
Rutherford class, mean (SD)	3.12 ± 0.05	3.81 ± 0.12	4.59 ± 0.34	21.89 (.000)***	Severe, mild to moderate > no POD
Rutherford class, n (%)				48.95 (.000)***	Severe > mild to moderate > no POD
0 asymptomatic	1 (0.2)	0 (0)	0 (0)		
1 mild claudication	6 (1.1)	0 (0)	0 (0)		
2 moderate claudication	224 (40.4)	23 (21.1)	1 (6.7)		
3 severe claudication	149 (26.9)	23 (21.1)	1 (6.7)		
4 resting pain	60 (10.8)	18 (16.5)	3 (20.0)		
5 minor tissue loss	99 (17.9)	37 (33.9)	8 (53.3)		
6 major tissue loss	15 (2.7)	8 (7.3)	2 (13.3)		
**2. Postoperative measurements**					
Duration of POD, mean ± SE, day	-	1.83 ± 0.10	6.93 ± 0.29	368.53 (.000)***	Severe > mild to moderate
CAM-ICU score, mean ± SE	-	1.86 ± 1.20	6.40 ± 6.09	187.62 (.012)*	Severe > mild to moderate
Length of surgery, mean ± SE, min	253.94 ± 3.34	290.37 ± 7.51	309.27 ± 21.60	12.17 (.000)***	Severe, mild to moderate > no POD
Length of ICU stay, mean ± SE, day	1.72 ± 0.06	2.16 ± 0.13	7.11 ± 0.36	109.25 (.000)***	Severe > mild to moderate > no POD
Death within 30 days, n (%)	3 (0.5)	1 (0.9)	0 (0)	0.31 (.86)	ns
Death within 90 days, n (%)	5 (0.9)	1 (0.9)	0 (0)	0.14 (.93)	ns
Death within the follow-up period, n (%)	56 (10.0)	11 (10.0)	1 (6.67)	1.67 (.43)	ns
Length of hospital stay, mean ± SE, day	13.61 ± 0.60	20.94 ± 1.35	29.82 ± 3.90	19.22 (.000)	Severe, mild to moderate > no POD

*ABI* ankle-brachial index, *SD* standard deviation, *SE* standard error, *POD* postoperative delirium, *CAM-ICU* confusion assessment method-intensive care unit.

*P < .05, **P < .01, ***P < .001.

The mean ABI values were significantly lower in the patients with severe POD (0.29) and mild to moderate POD (0.45) than they were those without POD (0.52) in a sequential manner (*P* < 0.001). Patients with mild to moderate (3.81) and severe POD (4.59) had a higher mean score of the Rutherford class than did those without POD (3.12) (*P* = 0.000). A significantly higher proportion of patients with severe POD (86.7%) and mild to moderate POD (57.28%) were categorized into higher Rutherford classes (≥ 4) than were those without POD (31.2%) (*P* < 0.000).

The overall mean duration of POD was 2.47 ± 2.81 days. The mean duration of POD (6.93 vs.1.83 days, *P* < 0.000) was longer and mean CAM – ICU score (6.40 vs. 1.86, *P* = 0.012) was higher in severe POD group than mild to moderate group.

The length of surgery (309.27 for severe, 209.37 for mild to moderate vs. 253.94 for no POD, minutes, *P* < 0.000) and hospital stay (29.82 for severe, 20.94 for mild to moderate vs. 13.61 for no POD, days, *P* < 0.000) were significantly longer in patients with POD than they were in those without POD. In addition, the mean number of ICU days was significantly greater in patients with severe POD (7.11) and mild to moderate POD (2.16) than it was in those without POD (1.72) in the order of POD severity (*P* < 0.000). During the study period, the number of patients who died was 68, and the number of patients who reached the last date of follow-up was 615. The mortality rate of study patients within 30 days, 90 days and the follow-up period (total mean 2156.66 days) were not affected by the presence of POD.

### Association between ABI and POD development

Table 3 shows the results of univariate and multivariate analyses of the variables that affect POD development in the study patients. Based on univariate analysis, the following parameters were positively associated with increased odds of POD development: age, CCI, length of surgery, Rutherford class and ASA mean values. The higher preoperative ABI significantly decreased the odds of developing POD (OR 0.08, 95% CI 0.03–0.21, *P* < 0.001). In addition, univariate analyses also showed occupational status and education were positively associated with increased odds of POD development.

**Table 3:** Univariate, multivariate and bootstrap-adjusted analyses of the variables associated with POD.

Variable	OR (95%CI)		
	Univariate	Multivariate	Bootstrap-adjusted
Gender, male	1.38 (0.79–2.40)	1.10 (0.60–2.00)	1.12 (0.46–1.67)
Age, mean	1.05 (1.02–1.07)***	1.04 (1.02–1.06)**	1.03 (1.01–1.06)*
Occupational status	0.48 (0.31–0.76)**	0.93 (0.54–1.59)	0.91 (0.53–1.57)
Education	0.83 (0.72–0.95)**	0.90 (0.77–1.04)	0.89 (0.76–1.04)
CCI, mean	1.26 (1.11–1.43)***	1.20 (1.04–1.39)*	1.19 (1.01–1.40)*
Length of surgery	1.01 (1.00–1.01)***	1.00 (1.00–1.01)*	1.00 (1.00–1.01)*
ABI, mean	0.08 (0.03–0.21)***	0.28 (0.10–0.78)*	0.32 (0.11–0.91)*
Rutherford class, mean	1.66 (1.42–1.93)***	1.44 (1.22–1.70)**	1.41 (1.19–1.67)*
ASA score, mean	1.93 (1.38–2.71)***	1.10 (0.72–1.69)	1.06 (0.69–1.65)

*OR* odds ratio, *CI* confidence interval, *CCI* Charlson comorbid index, *ABI* ankle-brachial index, *ASA* American anesthesiologist association.

*P < .05, **P < .01, ***P < .001.

In multivariate analysis, the following parameters were associated with POD development: older age (adjusted OR [AOR] 1.04, 95% CI 1.02–1.06, *P* < 0.01); higher CCI (AOR 1.20, 95% CI 1.04–1.39, *P* < 0.05); longer duration of surgery (AOR 1.00, 95% CI 1.00–1.01, *P* < 0.05): higher Rutherford class (AOR 1.44, 95% CI 1.22–1.70, *P* < 0.01); and decreased ABI (AOR 0.28, 95% CI 0.10–0.78, *P* < 0.01).

**Figure 2:**
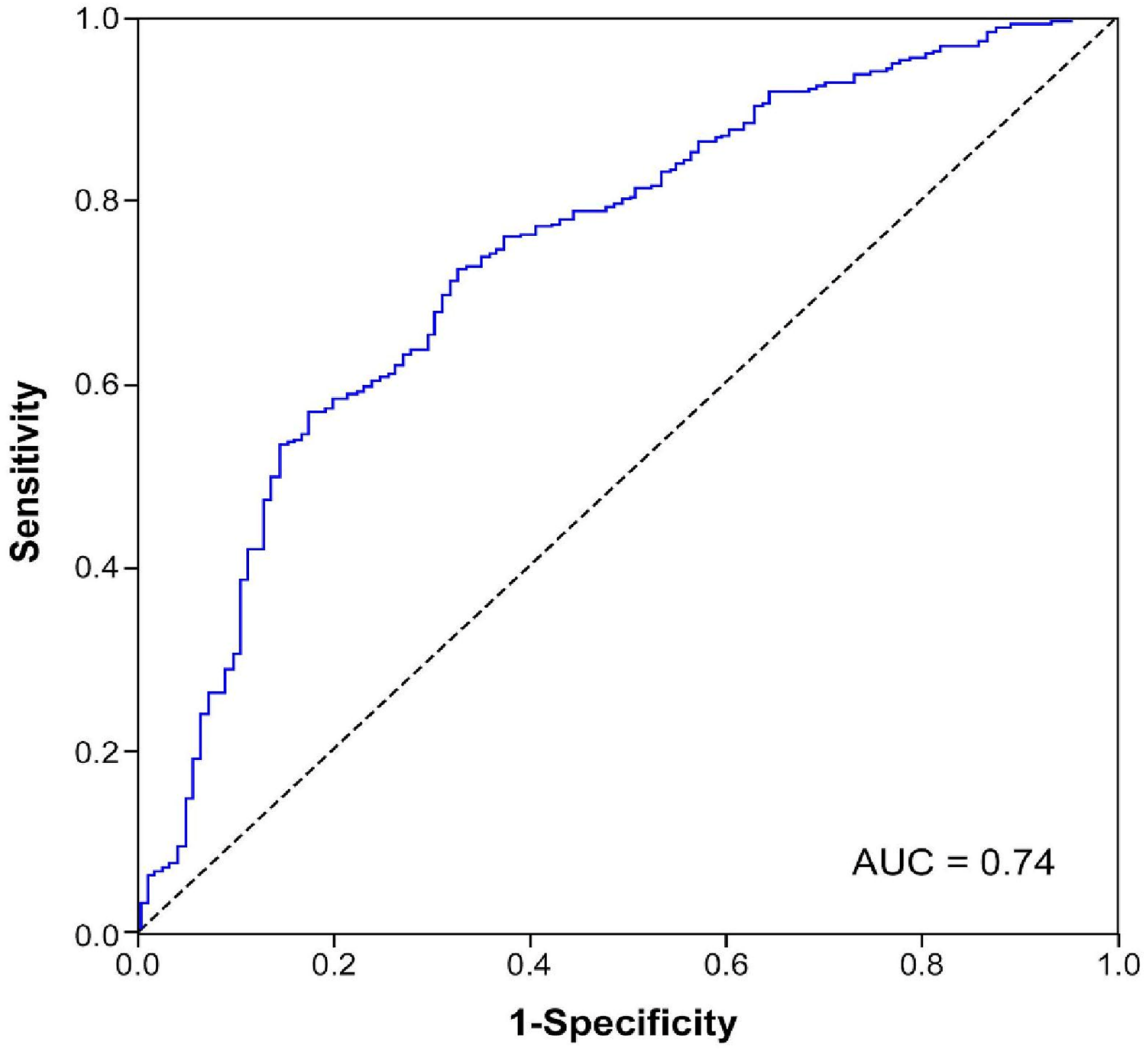
Receiver operating characteristic (ROC) curve of the Ankle-brachial index (ABI) for the prediction of postoperative delirium (POD) in PAD patients underwent peripheral arterial bypass surgery. Sensitivity (91.9%) and specificity (73.6%) at a cutoff of 0.35. Area under the curve (AUC) is 0.74.

In order to evaluate the effectiveness of ABI for estimating its association with POD in PAD patients who underwent arterial bypass surgery, we performed the ROC analysis (Fig. 2) The ROC analysis for preoperative ABI on POD (AUC 0.74, *P* = 0.0001) revealed that ABI below 0.35 was associated with development of POD. (with a sensitivity 91.9% and specificity 73.6%).

## Discussion

To the best of our knowledge, this is the first study to evaluate the association between ABI and development of POD in PAD patients who underwent elective leg arterial bypass surgery. Our study shows that PAD patients who subsequently developed POD had a significantly lower preoperative ABI than did those without POD.

Postoperative delirium is known to be an important contributor to poor medical outcomes and high socioeconomic burden^29,30^. Therefore, previous studies have attempted to identify the predictive factors of POD in various surgical patients^29,31–33^ and emphasized prevention.

PAD itself can impose POD risk due to narrowing of blood vessels along with hypoxemia^34^, which can cause insufficient oxygenation of the neurologic system and ultimately cause poor neurologic outcomes. However, prior studies that have addressed the risk of POD in PAD were limited. In such studies, PAD was often evaluated as one of preoperative comorbid conditions.

Therefore, our study is noteworthy because it was specifically conducted on a large PAD patient cohort in which we utilized ABI as a marker for identifying the high risk group of POD. In addition, the severity of POD based on the CAM-ICU score was taken into consideration for detailed analysis in this study.

The incidence of POD in our study was 18.3%, which was similar to that of previous studies, which reported an incidence of 14–39% after elective vascular surgery^4–6,35,36^. In this study, older age, unemployment, and more medical comorbidities (CCI, ASA score) were associated with POD and its severity. These results support previous studies, which found that the incidence of POD increased significantly over an age of 60 years^37^, with the presence of medical burden in the general acute setting^38^, and after vascular surgery^4^.

We also found that patients with more severe PAD were prone to develop more severe POD than were those with less significant PAD. A higher preoperative Rutherford class and lower ABI significantly increased the odds of developing POD. This is consistent with the results of Sasajima et al.^39^, who found that critical limb ischemia (Rutherford class 4–6) was an independent risk factor for delirium after bypass surgery. In particular, Rutherford classes 5 and 6 represent a certain degree of tissue loss that ultimately requires minor or major limb amputation. Visser et al.^4^ reported that amputation surgery was one of the risk factors that predict POD after vascular surgical procedures. One study found that as many as 61% of vascular patients developed POD after a major amputation^40^. A high rate of POD in patients with critical limb ischemia has been attributed to the inflammatory process, oxidative stress, and also by limitations of physical performance^4,37,41^.

Patients with POD also have an increased risk of poor outcomes, such as increased length of ICU and hospital stay, than did those without POD. These findings were consistent with those of previous studies^2–4,9,30,37^. However, the mortality rate in our study was not affected by the presence of POD. Although our results showed that a higher ASA score and CCI were associated with POD prevalence, it is difficult to conclude a relationship between POD and mortality without evaluating individuals’ medical risks more precisely.

There have been multiple efforts to identify the predictive markers of delirium, such as increased C-reactive protein (CRP), particularly with regard to a potential neuro-inflammatory etiology^33,42^. It has been hypothesized that surgical stress itself can increase inflammatory markers. This may be particularly important in vascular patients, who have chronic ongoing inflammation at baseline^42,43^. However, such inflammatory markers increase in response to systemic inflammation, rather than to a specific disease process.

The ABI is solely used to diagnose PAD, and its disease-specific diagnostic value has already been established^11,12^. In addition, the ABI is a relatively simple quantitative measurement^44^. In our study, both univariate and multivariate analyses showed that decreased ABI was associated with the development of POD after arterial bypass surgery. Lower ABI values were associated with more severe POD. ABI has already has been studied as a predictive marker of cognitive impairment in the past^11,45–47^. In a systemic review of the general population, one group reported that an ABI < 0.9 suggest an individual’s susceptibility to the development of cognitive disorders^11^. In an Edinburgh artery study, the ABI was predictive of cognitive impairment for up to 10-years of follow-up^47,48^. However, the study populations in previous studies were generally composed of elderly people with and without vascular disease (or specifically PAD). In addition, an ABI < 0.9 provides too broad a degree of PAD. Therefore, the optimal cutoff ABI of 0.35, which we suggested, can compensate for this limitation.

Although the occurrence of POD is multifactorial, ABI can be a good additional preoperative indicator to classify high-risk patients with other known risk factors, especially in a specific population with peripheral arterial disease. Identifying high-risk patients with this disease-specific test and considerable cutoff value is not sufficient for prevention of POD. However, in this specific population with peripheral arterial disease presenting low ABI, early recognition of high risk patients allows care providers to consider more prompt preventive measures by both non-pharmacologic and pharmacologic approach. In addition, patients with peripheral arterial disease are prone to undergo multiple vascular procedures hence increased the risk of future delirium. Although ABI would be considered as non-modifiable factor, early identification of high risk group using a disease-specific factor is important to monitor its association with POD continuously^49,50^.

## Conclusions

In PAD patients, the preoperatively low ABI is associated with the development of postoperative delirium. In contrast to the biochemical markers, ABI is disease-specific. Therefore, our results suggest that ABI within a certain range, is a good indicator to identify high risk group of POD in PAD patients.

## Limitations

Since our conceptual framework focused on PAD patients alone, our results are not necessarily generalizable to non-vascular patients. In this study, POD incidence is limited because it was only collected postoperatively in the ICU. We are aware of that not all facilities send patients undergo arterial bypass surgery for routine postoperative ICU care. Since the length of ICU stay itself negatively affect development of POD in general, our results might be limited in those settings. Future investigations are needed to evaluate delirium on the general wards, and for a longer period. Although surgical methods and pre-,peri-,and post-operative management did not vary by protocol over such a long study period, there are still changes in general medical environment that were not included for the analyses at this time. Although patients with abnormally high ABI were excluded from this study, those patients are also at risk for POD. It will be a good future work to use other diagnostic test or criteria than ABI to cover all patients regardless of arterial wall calcification. Lastly, this study was performed using data from a single-center database.

## Data Availability

The datasets generated during and / or analyzed during the current study are available from the corresponding author on reasonable request.
